# Participatory songwriting in a Belgian asylum reception center: addressing challenges through embodied strategies

**DOI:** 10.3389/fpsyg.2025.1602017

**Published:** 2025-07-09

**Authors:** Tina Reynaert, An De bisschop, Luc Nijs

**Affiliations:** ^1^Department of Art, Music and Theatre Science and Department of Social Work and Social Pedagogy, Ghent University, Ghent, Belgium; ^2^Department of Art, Music and Theatre Science, Ghent University, Ghent, Belgium; ^3^Department KASK-Conservatorium, HOGENT-University of Applied Sciences and Arts, Ghent, Belgium; ^4^Department of Social Education and Technology, Institute for Musicology and the Arts, University of Luxembourg, Esch-sur-Alzette, Luxembourg

**Keywords:** participatory music-making, songwriting, asylum reception center, embodiment, constraint-led approach

## Abstract

In recent years, asylum reception centers in the Global North have emerged as distinct settings for participatory songwriting activities. Despite the growing body of research on music facilitation within these contexts, a more comprehensive exploration of challenges associated with songwriting is required, alongside the development of both conceptual frameworks and practical tools to optimize participatory songwriting facilitation. This study employed an action research approach to critically examine and enhance the music facilitation process of *The Scratch Band*, a songwriting project in a Belgian reception center. By analyzing preparatory notes, transcribed and annotated video recordings of rehearsals, and a reflexive diary, challenges encountered during songwriting workshops were identified and strategies were designed strategies to address them. The findings reveal three key insights: (1) the researcher-facilitator faced place-based, person-based, and procedure-based challenges; (2) embodied strategies—including soundwalks, embodied musical brainstorms, timbral improvisation and moving lyrics—fostered participant-led creativity; and (3) these embodied strategies aligned with the constraint-led approach, suggesting its potential as a framework for conceptualizing practices and designing facilitation tools. This study demonstrates the potential of action research to reflect and improve music facilitation in participatory music practices. The results offer practical implications for music facilitators, highlighting the use of embodied music strategies to address challenges in songwriting workshops with applicants for international protection.

## 1 Introduction

In recent years, songwriting has emerged as a musical activity in asylum reception centers (Beckles Willson, 2021; Caruso, 2022a, 2022b; Farhan, 2023; Kenny, 2022; Mangifesta, 2019; Nicolaou et al., 2023; Pistrick, 2020; Weston and Lenette, 2016). In these reception facilities in host countries across the Global North, applicants for international protection find refuge during their asylum procedures (Kreichauf, 2018), as wars, conflicts, human rights violations, and climate change continue to compel countless individuals to cross international borders (International Organization for Migration, 2025; United Nations High Commissioner for Refugees, 2023). Although material assistance provided to applicants for international protection varies significantly across host countries (Ziersch and Due, 2018), reception facilities function as liminal spaces—environments characterized by uncertainty (Hartonen et al., 2022; O’Reilly, 2020). Living in these transient settings often amplifies post-migratory stressors and mental health issues, particularly for individuals who have already endured psychological difficulties due to prior traumatic experiences (Filges et al., 2024; Mulcaire et al., 2024).

Within these liminal spaces, songwriting can play a transformative role. It offers ways to amplify voices and shed light on the complexities of forced migration (Beckles Willson, 2021; Mangifesta, 2019; Pistrick, 2020; Farhan, 2023); express transnational identities through multilingual songwriting (Caruso, 2022a); and provide relief from the hardships of the asylum process while fostering a sense of community based on shared experiences (Caruso, 2022b; Lenette et al., 2015; Weston and Lenette, 2016).

Despite this positive impact, scholars have noted challenges when initiating music projects in asylum reception centers. Music facilitators struggle to gain access to these centers or to secure appropriate rehearsal spaces (Kenny, 2022). Participants, often dealing with mental health issues, may not prioritize music workshops, leading to difficulties in recruitment and inconsistent attendance (Beckles Willson, 2021; Kenny, 2022; Lenette and Procopis, 2016; Pistrick, 2020). The transient structure of asylum systems adds further complications: individuals are frequently relocated or leave due to changing circumstances (Kenny, 2022; Lenette and Procopis, 2016). Working in these contexts can also have an impact on music facilitators’ well-being (Lenette and Procopis, 2016).

Other studies have explored challenges related to music facilitation, here understood as “a structure for creative group work” aimed at “encouraging open dialog among different individuals with differing perspectives” (Higgins, 2008, p. 330). In terms of challenges, music facilitators frequently find themselves adapting their initial goals when workshops unfold unpredictably (Beckles Willson, 2021; Caruso, 2022a; Kenny, 2018, 2022; Pistrick, 2020). The multilingual character of these environments (Nicolaou et al., 2023; Pistrick, 2020) and the necessity to navigate different gender norms (Lenette et al., 2018) may also create challenges.

Yet, when it comes to examining music facilitation processes and challenges tied to specific musical activities—songwriting, for instance—the research remains sparse. Pistrick (2020) identifies a tension between his efforts to activate participants’ “sonic agency” (LaBelle, 2018) and the disempowering realities of life in asylum reception centers. Additionally, he notes that many participants are unfamiliar with the open-ended nature of co-creation, which prioritizes experiment. Beckles Willson (2021) highlights the challenge of fully understanding participants’ musical requests (e.g., in co-creating rap songs), which can be intensified by a lack of shared vocabulary for articulating musical elements.

Furthermore, most practice-based studies in asylum reception centers provide general implications for facilitating music workshops rather than tools to address songwriting-specific challenges. Kenny (2022), for instance, advocates for flexible, responsive methods that accommodate diverse needs. Flexibility may involve relinquishing rigid structures, allowing participants to help select repertoire, incorporating games or instrumental play, and sometimes centering workshops around songwriting. Similarly, Nicolaou et al. (2023) recommend non-verbal strategies—like expressive gestures—to enhance communication in multilingual contexts and suggest movement as a way to boost engagement.

While these insights are valuable, the limited scholarly focus on songwriting-specific challenges calls for deeper inquiry into the facilitation processes that shape and sustain songwriting practices. To respond to this call, we set up an action research project to examine a songwriting practice at a Belgian asylum reception center. Acting as a researcher-facilitator (RF), the first author critically examined her own practice, devised, and implemented strategies for its enhancement, and assessed both intended and unintended outcomes through iterative cycles of planning, acting, observing, and reflecting (Cain, 2008; Elliot, 1991; McNiff and Whitehead, 2005). She initially designed a series of songwriting workshops to support participants in song creation. In response to challenges that occurred during these early workshops, she developed and adapted strategies accordingly. This paper outlines the identified challenges and reflects on the various strategies implemented throughout the project. Notably, these strategies inadvertently integrated music and movement, creating embodied music activities. The benefits of this approach, as explored in this study, contribute to the growing body of research on embodiment, particularly how integrating movement with music can promote resilience among vulnerable groups (Nijs and Nicolaou, 2021). Ultimately, the paper proposes embodied strategies in participatory music practices as effective tools for enhancing autonomy, transcending language barriers and musical skill limitations, and fostering meaningful engagement with songwriting.

## 2 *The Scratch Band*: a participatory music project

### 2.1 Project description

*The Scratch Band* is a participatory music project in a Belgian asylum reception center and was initiated by Concertgebouw Brugge, a Belgian concert hall and the RF in this study. This organized encounter between a music facilitator and participants aimed to address musical and social objectives (Sloboda et al., 2020; Van Zijl and De bisschop, 2023). Musical objectives were actively involving residents of a neighboring Red Cross asylum reception center in musical activities through carefully planned songwriting workshops that enabled the co-creation of original compositions. As part of the broader *Raise Your Voice Festival*, organized by Concertgebouw Brugge, the initiative specifically aimed at addressing social objectives such as social inclusion and providing a platform for self-expression for people whose voice is often silenced. In a context where applicants for international protection are frequently positioned as passive recipients of aid and spoken about rather than with, the project aimed to reverse this dynamic by offering participants the opportunity to take the stage and perform (original) music on their own terms, opening up the possibility for what might be termed “sonic citizenship”: the assertion of presence, agency, and identity through sound, in the absence of formal political recognition (O’Toole, 2014; Mangifesta, 2019). These workshops were designed as “sites for experimentation, creativity, and group work” (Howell et al., 2017, p. 605) and are situated within an “interventionist” framework that strives to connect with individuals facing challenging circumstances, using music-making as means to foster personal growth and enhance well-being (Howell et al., 2017). The project comprised fifteen 3-h workshops held on a weekly basis between August and late November 2024 at the asylum reception center.

The series of workshops concluded with a final performance in the chamber music hall of Concertgebouw Brugge, where the musical output was performed. In this project, the RF is a pianist and experienced music facilitator in asylum reception centers. Assuming the roles of both music facilitator and researcher enabled her to adopt an insider’s perspective (Frederiksen et al., 2021).

### 2.2 Preparatory phase and workshop structure

Prior to the start of the workshop series, the RF visited the reception center on two occasions. The first visit took place during an open-door day, 4 months before the project’s launch. This visit offered an opportunity to explore the reception center, meet staff and residents, and engage in informal conversations. A second visit took place shortly before the project’s start to meet again with the center manager about the coordination of logistical aspects and to have conversations with the residents about their musical interests; most of them showed the music they enjoyed via their phones or talked about earlier musical experiences. In the weeks before the workshops, staff informed residents about the music workshops through flyers in various languages and through short conversations. When the workshops began 2 weeks later, only new people showed up, and more participants joined in the next weeks.

The first workshops took the form of group jams in which five to 20 participants were engaged collectively. In response to participants’ suggestions and differing needs, workshops were structured into distinct segments: a group of four Arabic-speaking men who preferred working together; a group with four children meeting in the early hours after school; a women’s group and a concluding open session for anyone who wished to join in a collective jam, sometimes up to 10 participants. Throughout the workshop series, 21 participants with ages ranging from 8 to 55 engaged, representing diverse cultural and linguistic backgrounds. An overview of the participants demographic information is provided in Table 1.

**Table 1:** Participant demographics.

Variable	*N*
Age	
8–18 years old	4
18–55 years old	17
Gender	
Male	14
Female	7
Country of origin	
Syria	9
Kurdistan Irac	4
El Salvador	1
Palestine	2
Eritrea	2
Cameroon	1
Afghanistan	1
Tunisia	1

**Figure 1:**
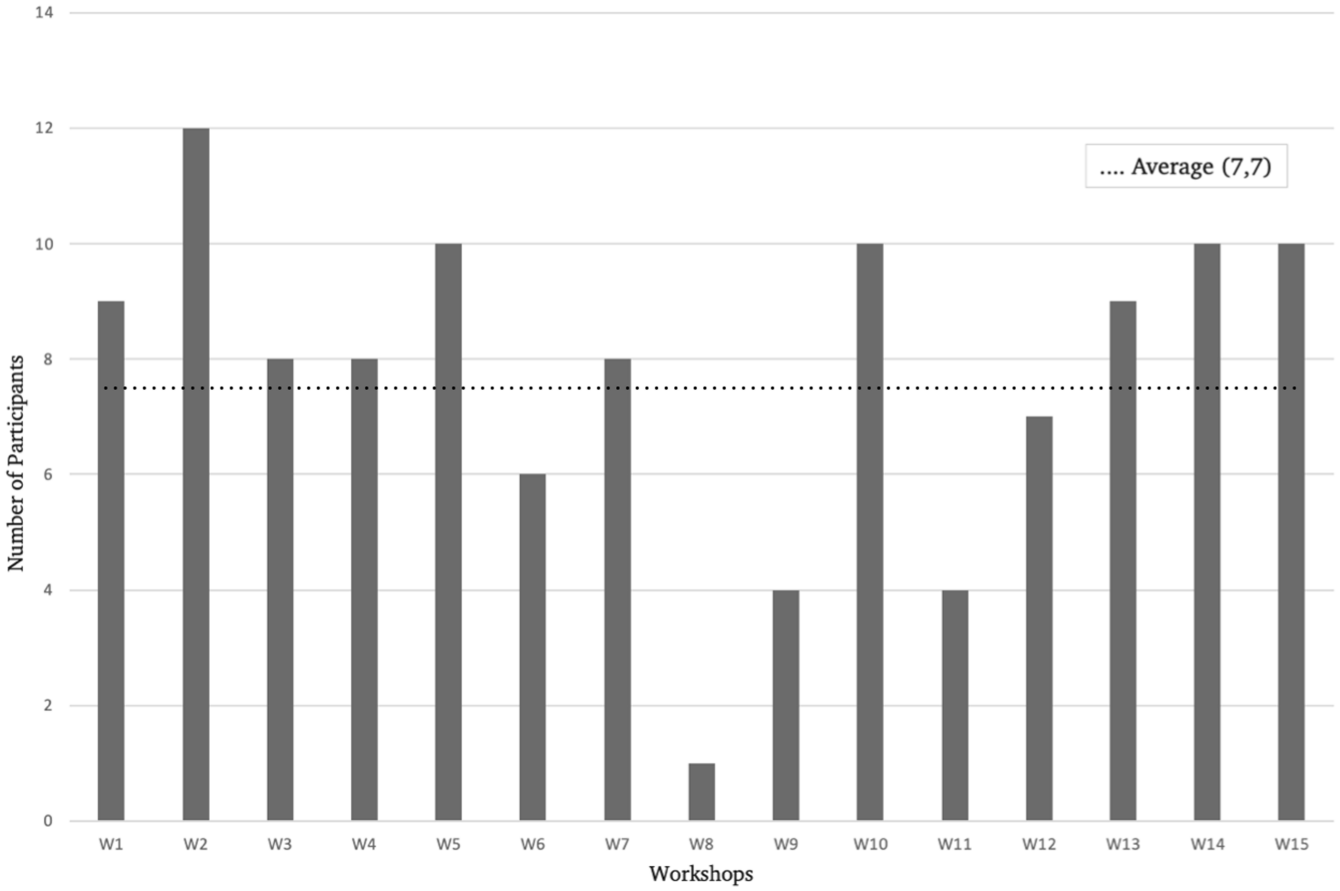
Overview participant attendance.

Participants were free to attend the weekly workshops or join the final concert. As a result, some individuals engaged in the rehearsals but chose not to take part in the public performance. Over the course of the four-month project, the composition of the group remained fluid, with participants frequently arriving or departing due to changes in their residency status and some participants arriving later in the workshops due to work or other commitments. Ultimately, 10 participants joined the final concert performance. An overview of the participation numbers per workshop (W1–15) and the average number of sessions attended per person gives more insight in participant attendance (see Figure 1).

### 2.3 Pedagogical premises

The design of the workshops, as described more in detail in 2.4, was informed by a set of pedagogical premises derived from two primary resources: (1) practical tools and implications identified in previous research on music facilitation in asylum reception centers (e.g., Kenny, 2022; Nicolaou et al., 2023), and (2) theoretical concepts drawn from the broader academic literature on participatory music or community music (e.g., Camlin, 2015; Higgins, 2012; Higgins and Campbell, 2010; Howell et al., 2017).

A first premise was to conceptualize the workshop as what Higgins (2012) calls “act of hospitality,” starting with “a welcome (given by those that name themselves community musicians) to those who want to participate in active musical doing” (p. 142–143). Hospitality, in this context, means opening the workshops to all—welcoming diverse backgrounds and musical skills, without auditions or restrictive selection criteria. This approach is grounded in the belief that all individuals have an “innate capacity and lifelong potential for musical engagement and creativity*” as well* as a fundamental right to create, participate in, and enjoy music (Howell et al., 2017, p. 611).

A second premise involved adopting a responsive facilitation style. This entailed engaging participants in (1) the selection of musical activities, tailored to their preferences at a given moment (Kenny, 2022), and (2) the creative process itself, where spontaneous ideas and actions were embraced and incorporated (Nicolaou et al., 2023). The workshop served as a dialogical space, drawing on “the collective wisdom of the group to shape, at least in part, the creative direction of the work*”* (Camlin, 2015, p. 239). The musical processes are approached as “a trail of no mistakes*”* (Higgins and Campbell, 2010, p. 8), emphasizing the value of diverse and dynamic musical pathways that music facilitators and participants can explore together. A third premise was the use of non-verbal instructions—through expressive gestures or by offering examples through music—as research shows that extensive verbal instruction in a new language is ineffective in multilingual settings (Nicolaou et al., 2023).

### 2.4 Musicking activities

The above-mentioned premises inspired the design of an initial set of musicking activities, with musicking understood here as “to take part, in any capacity, in a musical performance, whether by performing, by listening, by rehearsing or practicing, by providing material for performance, or by dancing” (Small, 1998, p.9).

The RF chose to start with jams, as it is a way to explore participants’ musical backgrounds and interests while generating creative ideas. To use fewer verbal instructions, the RF started playing a groove, chord scheme, or (Arabic) repertoire she learned in previous projects to inspire and encourage participants to follow by example. Participants could join the circle, bring in songs they knew, or created lyrics and musical elements on the spot. By using expressive gestures to change the volume, or by clapping rhythms the RF gave the jam more direction. When participants entered, they could pick an instrument, or the RF offered them one. They could listen or dance. All individuals affiliated with the asylum reception center were welcomed, also staff. The latter were unable to participate as they were too busy with their daily tasks.

Jamming workshops were complemented with more intimate songwriting workshops in which participants and the RF wrote lyrics together and put them into music. Songwriting is understood here as the “lyrical expression of human thoughts and emotions through melodic, rhythmic, and textual ideas” (Cohen and Wilson, 2017, p. 545). This is more language-based in smaller groups, using translating apps to support creative negotiations. There are various reasons for choosing songwriting as an activity. First, it can be considered as an accessible activity. The voice—whether spoken or sung—is something most participants already carry within them. In contrast, musical instruments, or the skills required to play them, are not as commonly available. Second, a large body of literature on the use of songwriting in participatory music practice shows its many benefits. Engaging participants in songwriting (1) leverages “their innate capacity for being creative and playful in their day-to-day life” (Mani, 2022, p. i39); (2) provides a platform for self-expression and enjoyment (Cohen and Wilson, 2017); (3) helps to amplify voices that are forgotten (Birch, 2023; Gallo and Kuchenbrod, 2022; Mani, 2022); (4) offers opportunities for “reflecting on past experiences” (Cohen and Henley, 2018, p. 166); and (5) by sharing their experiences both within and beyond workshop settings, participants can “challenge dominant negative narratives” (Hess, 2018, p. 7). Third, in the case of this project, it provided the opportunity to create a musical output for the final concert in Concertgebouw Brugge. However, in alignment with the principle of responsive facilitation, it is important to highlight that songwriting activities were not imposed on the participants. Songwriting was not considered as more effective than other musicking activities, and those who preferred performing existing repertoire were invited to share their musical preferences and perform these pieces during rehearsals and concert. For individuals less inclined to engage in musicking, alternative forms of participation were offered, such as engaging in photography workshops or creating visual works that could be used in the project and concert. Positioned as a dialogical space, the project allowed participants to make decisions on various aspects of the songwriting process. These included (1) working in participant-facilitator dyads or smaller subgroups, (2) selecting the topics and languages of the lyrics or the objects about which lyrics were made, (3) exploring and determining the musical accompaniment, if it was needed and (4) deciding how the songs or lyrics would be performed, with participants being free to modify choices at any stage. Exercises that were used were writing lyrics based on objects or topics selected by participants, and improvisational dialogs in which participants found topics to use in their lyrics through jamming.

## 3 Methodology

### 3.1 Action research model

This study is a form of Action Research (AR), as it is research undertaken by practitioners (e.g., teachers, music facilitators) who aim to improve their own practice (Elliot, 1991). Action research thus resonates with the terms such as “teacher research” (Lytle and Cochran-Smith, 1992) “practitioner research” (Middlewood et al., 1999) and “self-study” (Loughran, 2005), practitioner-led or practitioner-based research (McNiff and Whitehead, 2005). In seeking answers to improve their own practice, action researchers investigate their own practice, plan and carry out interventions to improve it and evaluate the intended and unintended consequences of these interventions, interrogating data in order to ground their evaluations in evidence. They reflect on each stage to generate new plans, thus starting the cycle again (Cain, 2008). As the focus of this action research is to improve the facilitation of a participatory songwriting project, the first author acted as researcher-facilitator. Although this method is primarily researcher-led, the facilitator can make modifications based on shared feedback from collaborative members of the group (Elliot, 1991). Indeed, while participants of the music project were not involved as co-researchers throughout the project, there was a continuous dialog with the participants. During rehearsals, the RF regularly sought participants’ feedback—asking how they experienced the activities, what drew them in (or caused them to withdraw), and what challenges they encountered. These conversations were documented in her reflexive diaries and were instrumental in shaping the subsequent cycle of the action research. Therefore, participation in this study refers to the active involvement of individuals seeking international protection in musicking activities that were shaped through co-creation, i.e., a dialogic process in which musicians—both trained and emerging—collaboratively share, reflect upon, and shape the musical experience (Higgins, 2012). This conception aligns with Matarosso’s (2019, p. 19) definition of participatory art as “the specific and historically recent practice that connects professional and non-professional artists in an act of co-creating art.”

### 3.2 Data collection and analysis

For each workshop (*n* = 15), three types of data were collected. The first data set consists of the preparatory notes (PN), in which different actions that were planned for each rehearsal are written down. The second data set includes the transcribed and annotated video footage (TV) of the workshops. The video footage was recorded with a free-standing camera placed in the corner of the music room at the asylum reception center. Dialogs during the music rehearsals were transcribed, and non-verbal elements important for the analysis were annotated in italics in the transcripts. As this analysis focused specifically on songwriting activities, segments of the rehearsals involving other forms of musicking (such as repertoire performance or listening activities) were acknowledged but not fully transcribed. Instead, attention was directed toward musicking activities that involved songwriting. To differentiate between songwriting and less relevant moments centered on reproduction, the transcripts were segmented into scenes (“s”), with a new scene beginning whenever a shift in musicking occurred or when new participants entered the room. In the result section, for instance (TV1: s1) refers to the first scene in workshop 1. The third data set comprises a reflective diary (RD), containing immediate observations and reflections after every workshop.

**Figure 2:**
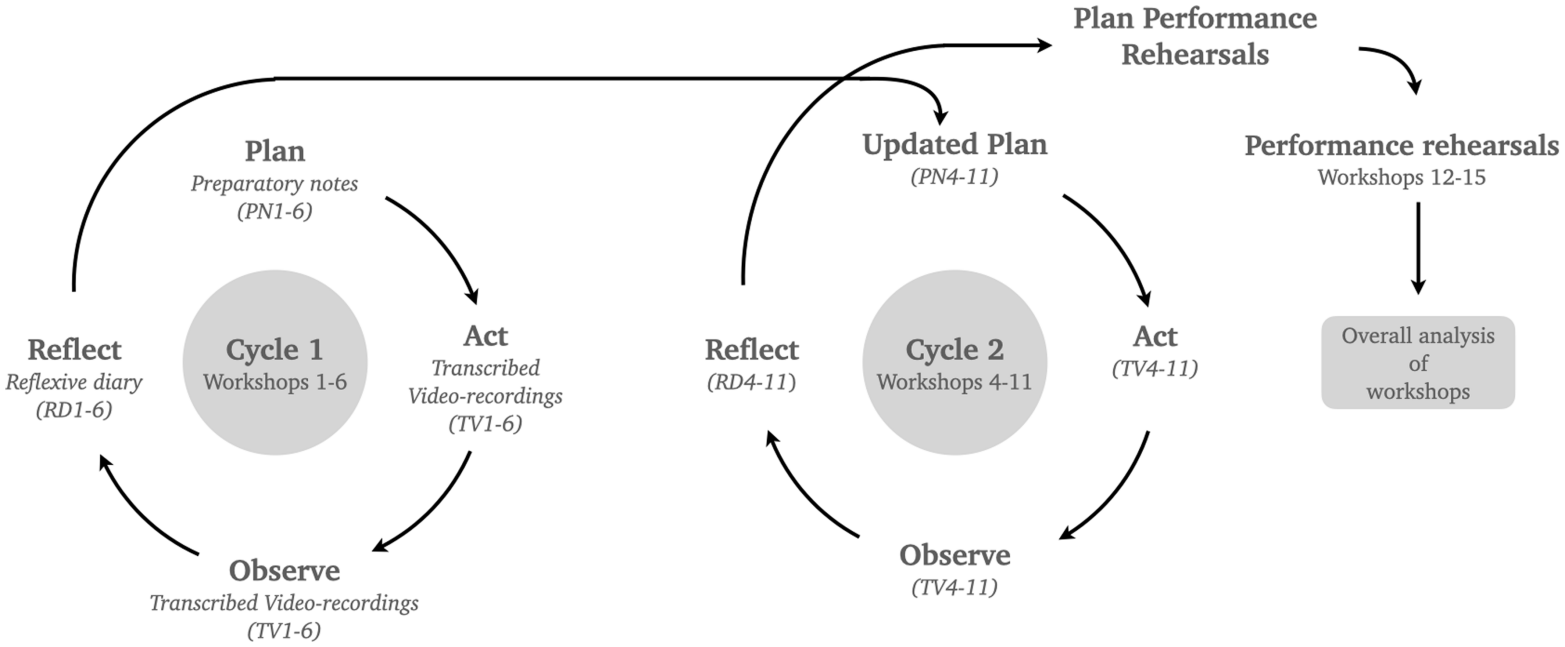
Based on an analysis of preparatory notes (PN), transcribed video recordings (TV) and reflective diaries (RD), two iterative action research (Plan-Act-Observe-cycles) led to the plan for the performance rehearsal and, ultimately, to an overall analysis of the process.

While the RF was actively engaged in executing planned activities and reflecting on their implementation after each workshop, the cyclical process of this action research spanned a longer period. After identifying challenges, multiple rehearsals were required to implement appropriate strategies, as participant attendance was sometimes inconsistent. As a result, this project only contains two action research cycles (see Figure 2): a first cycle involving the first six workshops in which an initial set of musical activities was implemented (jamming and songwriting, see 2.4) and challenges that occurred in response to these musical activities; a second cycle referring to workshop 4–11 in which the RF started to address challenges from cycle 1 by implementing embodied strategies. There was an overlap between cycle 1 and 2 because some challenges are connected to participants that joined the project later, while the RF already started to address other challenges that appeared in earlier workshops. The action research cycles included only workshop rehearsals 1–11, as the focus lay on the changes in the facilitators’ approach to deal with the encountered challenges. Although the final rehearsals (workshops 12–15) built upon these changes, they did not constitute a distinct research cycle.

Data analysis occurred at two moments throughout the project. First, between workshops, the RF reviewed video recordings of the rehearsals (TV) and revisited relevant passages in the reflexive diary (RD). During this process, the RF identified and documented instances of challenges encountered during rehearsals. These observations informed the development of the preparatory notes (PN) that aimed at improving the structure and effectiveness of the subsequent workshop. This approach allowed both analyzing the data for research purposes and directly applying the findings to the participatory music practice.

The second phase of analysis occurred after the completion of the workshop series. At this point, all collected datasets were analyzed using thematic analysis. Thematic analysis is a qualitative method that is used to identify, analyze, and interpret meaningful patterns (themes) across data (Braun and Clarke, 2006). In this study, implementing this data analysis method involves identifying recurring patterns and emergent themes to distinguish the various challenges encountered throughout the songwriting process. Moreover, it can be applied—as in this case—when different types (text data, video data) and large datasets are used (Braun and Clarke, 2012, 2013).

To streamline the analysis, all datasets were compiled into a single document, organizing the data chronologically. The structure followed this sequence for each workshop: preparatory notes (PN), transcripts of verbal interactions (TV), and reflexive diaries (RD) (e.g., PN1, TV1, RD1, PN2, TV2, RD2, etc.). This structured format allowed the RF to track the progression of the songwriting process and analyze patterns of action and reaction over time.

As thematic analysis was used to identify challenges in the songwriting practices, all sentences referring to such challenges were extracted and labeled based on their respective location (e.g., RD5 or TV6: s1). After this preparatory step, the analysis proceeded through several stages, following Braun and Clarke’s (2006) framework. The first phase involved an immersive reading of the dataset, where RF noted potential significant codes. In the second phase an inductive coding process was employed to systematically code all passages related to challenges. In the third phase, initial themes were identified by examining relationships between codes. The fourth phase involved reviewing these preliminary themes to assess their coherence with the passages concerning challenges. In the final phase, the themes representing the different categories of challenges were presented in the results section. To further investigate the strategies that were implemented, the datasets were examined in greater detail. Specifically, all strategies implemented in response to identified challenges were compiled and labeled according to the corresponding workshops in which they took place. By revisiting the preparatory notes associated with each strategy, the RF analyzed the reasoning behind its design and implementation. Additionally, the reflexive diaries were re-examined to assess the perceived impact of these strategies on the music facilitation process.

### 3.3 Ethics

Ethical clearance to conduct this research was obtained from the the Ethics Committee of Ghent University, with particular attention to the context-sensitive nature of the study. During the initial phase, potential participants received detailed information regarding the music workshops and the associated research via an information letter in English. Written informed consent was obtained for participation in the research project and for permission to be filmed. Participants were explicitly informed that they could engage in the music project without participating in the research and could withdraw from the study at any time. After each rehearsal, video recordings were immediately deleted from the physical storage device and securely transferred to a locked online repository managed by the involved university. To ensure participant anonymity, pseudonyms were used to identify individuals in the transcribed and annotated video recordings.

### 3.4 Statement of positionality

While leading and actively engaging in the music project, the RF critically reflected on her positionality. Methodologically, she considered how her identity as a white, middle-class, European academic in Belgium intersected with the feelings of boredom and unease expressed by participants that also want to study or work, and how this could influence interactions with participants and create power imbalances (De Martini Ugolotti, 2022). As a RF without a migration background, and with a keen awareness of her white privilege, the RF recognized that her social, cultural, political, and economic capital were not available to many participants due to their illegal status, enforced poverty, limited access to rights and paid work (Kenny, 2022). Moreover, conducting research with applicants for international protection positioned them as “other” and merely acknowledging researcher biases is not sufficient (Kenny, 2021). Therefore, as this is a research project about designing effective participation in songwriting workshops, the RF considered participants’ perspectives on songwriting activities to shape the design of the workshops. If participants had different interests or musical objectives, the workshop design was adapted accordingly. Additionally, the RF informed participants that she was not a staff member of the asylum reception center or a state officer deciding on their asylum application (Beckles Willson, 2021; Kenny, 2022). The RF clarified her role as a musician and music facilitator, with the primary goal of creating new music collaboratively. She also informed participants that her role as researcher was to address challenges in songwriting activities, which eventually would mean adapting a responsive facilitation style.

## 4 Results

### 4.1 Challenges in songwriting workshops

Thematic analysis indicated that place-, participant- and procedure-based challenges occurred in this songwriting project (see Table 2).

**Table 2:** Overview of challenges in songwriting workshops of *The Scratch Band*.

Place-based challenges	Person-based challenges	Procedure-based challenges
Noisy room Constant passage Verbal interruptions by outsiders	Different (musical)interests Limited inspiration Limited musical skills Language barriers in performance Language barriers in process Mental health issues Interpersonal barriers	Reluctance to testify Assistance with problems Forced departure

The first category of challenges related to the setting of the music room within the asylum reception center, constituting place-based challenges. The music project was conducted in a room that served as a living room, children’s playroom, dining area, and entertainment space, and the constant noise and passage were challenging for all those involved (RD1; RD2). Staff members and other residents of the center interrupted the music rehearsal to ask questions or start a dialog (RD3). Moreover, when mothers participate in rehearsals, they are mostly accompanied by their children due to the lack of available childcare (TV1: s1; TV2; TV3: s3; RD3).

The 19^th^-century building is originally constructed as the residence of a wealthy family of five to seven people, possibly including domestic staff. Today, that same structure accommodates over sixty individuals: men, women, children, and infants. The central living room is where music rehearsals are held, but also where meals are eaten, conversations unfold, children play, and people pass through at all hours. I got overwhelmed very easily, and participants as well, by the slamming of doors, people passing or eating, and toddlers banging on all instruments they can find (RD2).

The vibrant nature of this multifunctional room at times summoned a party-like energy, spontaneous and uncontained, which is typical for jamming workshops. New people sometimes entered, drawn perhaps by the music or mere curiosity (RD1; RD3). This lively openness also complicated the music rehearsals, sometimes interfering with trying to cultivate a safe, focused space for songwriting.

A second category of challenges concerned specific aspects of participants, such as (1) different (musical) interests, (2) limited inspiration and musical skills, (3) language barriers (4) mental health issues, and (5) interpersonal barriers.

Some participants displayed little interest in writing song lyrics and creating new songs. One example is a resident that expressed skepticism about the role of storytelling in songwriting, suggesting that personal issues are private matters and writing about his lived experiences or personal challenges might cast him in the role of a victim. Additionally, he prioritized work obligations to support his family, which prevented him from attending the rehearsals and concert (RD9). Some preferred other activities, such as engaging in sports (RD9) or being creative with visual media, including drawing and painting (RD9). Other participants noted that their musical interest lay elsewhere (TV1: s15; RD1; TV3: s5; TV4: s7; RD9). One participant, for example, preferred to perform repertoire that was already familiar to him:

F: I play some chords, and you improvise.

P: me? How, like freestyle? No no no

F: Sing about what you did today (*participant shakes his head and laughs*) just try.

*(participant is searching for a correct pitch to fit the chords; facilitator gives an example)*

P: I do not wanna sing this

F: Do not be scared!

*(participant tries to hum some notes)*

P: it’s a sad one that one he!

F: you want happy song? *(facilitator changes the chords; he tries again)*

P: just, let us sing another song, I do not wanna sing this, ok!

F: So you like to sing something that already exists and that you know?

P: Yes! Something jazz, like Fly me to the moon (TV1: s15)

Among those interested in songwriting, many struggled with the creative process, particularly the “blank canvas” of generating new lyrics or musical ideas. When encouraged to write lyrics or deliver ideas, participants had limited inspiration and relied heavily on the RF for direction (TV1: s1; RD1; TV2: s3, 5; TV3: s1). Additionally, most participants lacked formal musical education and had limited experience in music creation or performance. Apart from one individual, few participants could play an instrument, and for the majority, the project marked their first experience engaging in musical jams, as organized in cycle 1 of this action research project. Tasks such as improvising within specific keys or meters were particularly demanding, and participants struggled to maintain pitch while singing lyrics or to transform lyrics into melodies (TV1: s4; RD1; TV2: s3; TV4: s3,6; RD4; TV6: s1). The limited musical skills of some participants only became apparent once the workshops started. This was partly because they were not present during the initial preparatory work or only arrived after the start of the workshop series. Although community dialogs in the preparatory work phase could reveal their musical interests, it was only through active engagement in musicking activities that their actual musical skill levels could be assessed. Consequently, the RF had to adopt a role akin to a music teacher, providing instruction on rhythms, addressing technical issues such as pitch and timing, and correcting execution errors (TV1: s4; TV4: s1; RD4; TV6: s1). The following excerpt from the RF’s reflexive diary explains why she did not consider separate music instrument teaching sessions – as it occurs in similar projects with untrained and inexperienced participants:

Learning to play an instrument and improvise requires time and continuity—neither of which were available in this short-term project. Being the sole music facilitator further complicates the case. Given the upcoming concert performance at Concertgebouw Brugge, I need to explore alternative methods that focus on participants’ existing strengths, aiming for an inclusive performance that was both achievable and satisfying for everyone involved (RD4).

Language barriers further compounded these difficulties throughout the project. When participants preferred to write lyrics in Dutch to accommodate Dutch-speaking audiences, they faced considerable challenges when performing these texts. Limited Dutch proficiency hindered their ability to read and articulate lyrics fluently, and issues with pronunciation and comprehension further complicated the process (TV4: s3; RD4; TV5: s3; RD5). Similarly, when participants preferred to perform in their native languages, such as Arabic, Tigrinya, or Kurdish, significant obstacles emerged in refining lyrics and melodies as the RF did not know these languages (TV4: s3; RD4). Moreover, the absence of a shared language made it difficult to negotiate creative decisions during the songwriting process, despite the use of translation apps (TV4: s4; RD4; RD5).

Interpersonal barriers relate to underlying tensions between individuals, for various culture-related and gender-related reasons, but sometimes also for unknown reasons. During music rehearsals, male participants consistently attended in the company of others who spoke the same language or came from the same country, often performing repertoire they shared culturally. For reasons that remain unclear, collaboration with individuals from other ethnic backgrounds appeared limited or was avoided (TV3: s2; RD5). These interpersonal dynamics also intersected with the concerns of woman as they did not feel comfortable when the rehearsal space contained mostly men. Also, for Muslim women and mothers, participation in mixed-gender songwriting workshops was considered inappropriate:

Madam, I want to say, if I must sing or dance, then I cannot do it together with other men. Only with women—no problem. It is not right to be together with men like that. Why else would I wear a hijab? (TV2: s11).

In addition to interpersonal barriers, participants encountered a range of post-migration stressors, which significantly impeded their capacity to focus on songwriting (RD4; RD6). Mental health struggles, including experiences of depression and anxiety, further contributed to irregular attendance (RD6) and participants’ withdrawal from the project (RD4). While specific reasons for disengagement were not always provided, it is possible that personal circumstances and psychological distress hindered their motivation and ability to participate fully. Songwriting may have had the potential to evoke traumatic memories, yet participants did not articulate such concerns directly. In some cases, they simply stopped attending without offering any explanation (RD3).

A third category involved procedure-based challenges that refer to challenges that arose due to participants’ enrolment in pending asylum procedures. Participants perceived songwriting about lived experiences or life in limbo as potentially unsafe. Although participants expressed criticism regarding reception facilities, food, and general management of the center outside the music rehearsals, they were hesitant to translate these critiques into song lyrics (TV3: s2; RD3). There was a concern that such creative expression—if interpreted as criticism of their current conditions—might negatively affect their asylum applications (RD10). Moreover, they feared that voicing dissatisfaction might lead to eviction or even imprisonment, as was the norm in their countries of origin (RD10). Sometimes, participants were reluctant to testify, but did not give a reason for that (TV3: s2; RD3). One participant voiced doubt about the usefulness of expressing complaints at all. Despite the difficulties they faced, he emphasized that the bed, bath, and bread principle represented their final refuge, and that they should be grateful not to be homeless or living in precarious circumstances like those they had endured in previous camps (RD9). Their life in limbo also hindered the rehearsals, as participants asked assistance with personal and practical challenges, including support in translating important documents (RD9; TV10: s4). Furthermore, several participants had to leave the reception center over the course of the four-month project, due to changes in their procedure or negative asylum outcomes, which prevented them from continuing their involvement in the project (RD6; RD9; RD14).

### 4.2 Embodied strategies in songwriting workshops

To address above-described challenges, the RF designed and introduced strategies to enhance the effectiveness of facilitation in a second cycle of the action research (see Table 3). The strategies used in this study were (1) soundwalk, (2) vocal and bodily improvisation in favor of brainstorming, (3) timbral improvisation based on themes or guided by graphic scores, and (4) embodied engagement with lyrics through movement.

**Table 3:** Different challenges and the corresponding strategies to address them.

Challenges	Strategies
Place-based challenges	
Noisy setting Constant passage	Soundwalk: composing with noise
Participant-based challenges	
Limited inspiration	Voice and body improvisation: brainstorm Soundwalk: explore ideas
Limited musical skills	Timbral improvisation based on a theme Timbral improvisation using graphic scores Soundwalk: participants as composers Moving lyrics instead of or in addition to speaking/singing
Language barriers in performance	Moving lyrics instead of speaking
Procedure-based challenges	
Reluctance to testify	Moving lyrics instead of speaking Timbral improvisation Soundwalk

Addressing place-based challenges such as constant passage and noise, was difficult, as there were no other rooms in the reception center to organize the workshops. Nevertheless, the RF proposed organizing a sound walk which is an embodied way to explore the environment. While walking, participants would select noises and sounds, and attribute meaning to these noises. In this way, the RF would open a dialog with participants about the noises they hear every day and to explore the possibilities of creating something with noise. The selection of noises would be guided by a series of questions such as: “Which sounds do you hear daily?” “Which sounds bother you?” “Where do you stay, and what does that place sound like?.” These sounds would be compiled into a sound map of the asylum reception center. In this way, another perspective on noise, namely as something we can use to construct a music piece, rather than as a sole burden, could be generated (PN8).

On the day of the soundwalk, only one participant engaged in this activity. He selected noises and guided the RF, while sharing and explaining what these sounds meant for him (TV8; RD8):

He recorded footsteps on the stairs and the sharp slam of a door—sonic markers of the constant movement and transient presence of a large group of residents. The rhythmic striking of a boxing ball symbolized a personal outlet for processing anger and frustration. The subtle whir of his new bicycle wheel, expressing pride in finally owning a bike of his own. The assistant calling his name through the microphone, asking him to come to the reception. Lastly, the overlapping music played in the dining room, where individuals listened to YouTube videos without headphones, resulting in a sonic tapestry reflecting diverse musical styles (RD8).

As the afternoon passed by, he selected over 20 sounds and provided them with stories and background information for me to understand how his daily life looked like (RD8). Ultimately, he decided to program the final sound map during the concert performance to inform the concert audience about how the center sounded and raise awareness about the lack of silence, which was a concern he shared with other residents in the center (RD12).

A second category of strategies aimed at addressing person-based challenges, such as limited inspiration. Rather than focusing on writing lyrics and then setting them to music, participants were invited and encouraged to express their ideas through movement, sound creation, and vocal improvisation, drawing inspiration from their own drawings (PN3) or memories (PN6). These embodied musical brainstorms generated a wealth of new ideas, which had not emerged in earlier activities in which they were invited to just write or improvise a text (RD3; RD6; RD8). One example involved a collaborative exercise with a group of four participants who were asked to create a drawing depicting their current living space within the asylum reception center, with prompts such as “What does your current home look like?.” This exercise allowed gaining insights into how participants experienced the asylum reception center, culminating in the composition of the song “Vlamingstraat 55” (RD3).

On their drawing you see the large garden, and a building next to it. They told me they liked the plants, flowers and trees that formed a city garden. They added how they were confronted with an abundance of French fries, almost every day, but also watermelon and other more healthy fruits. Although the building looked like a normal family house with house number 55 on it, they told me that for every problem they had, they needed to go to the administrative office, to ask help or permission to do something (RD3).

This drawing, accompanied by words and brief sentences, served as a starting point for dialog, brainstorming, and lyric development. Through vocal improvisation and body percussion, participants found their own ways to express the elements present in the drawing (PN3; TV3: s2, 4; TV4: s4; RD4). Participants vocalized the address of the reception center, expressing each syllable with individual expression—some uttered it quickly or with high pitch (high, low, heavy, wide), others accompanied their speech with bodily gestures such as clapping or stamping. At times, they even mimicked the local West-Flemish accent, playfully engaging with their surroundings. They continued to use their voices and bodies to emphasize words like “large garden,” “plants,” “trees,” “flowers,” as well as phrases like “tasty food,” “watermelon,” and “always fries” evoking sensory impressions of their environment. In a subsequent scene, participants engaged in rope-skipping—an activity from their daily routine—while chanting sentences such as: “When we have a bleeding nose, to the reception,” and “When we have an aching ear, to the reception.” These performative lines subtly pointed to the institutional routines that shaped their current living conditions, distinguishing the reception center from the intimacy of a private family home. In another session, the same group of participants was invited to depict the behavior of asylum center staff using their bodies and voices (TV6: s2). They imitated staff members’ vocal timbres, gestures, and common phrases used in their interactions with the residents of the center. This exercise expanded participants’ creative engagement and expression (RD6) and contributed further to compose “Vlamingstraat 55,” as participants shared additional insights about interactions with staff (RD6; PR7; TV7). Similarly, the soundwalk —described earlier in this result section— engaged one participant to record sounds from his daily environment. In earlier songwriting workshops he had struggled to find topics to write about. During the soundwalk he was a composer with a lot of inspiration, as he could directly draw on sound material with which he was confronted daily (PN8; TV8).

A third category of strategies addressed the limited musical skills of the participants. Rather than focusing—as in cycle 1—on tonal improvisation and group jamming, which typically involves alignment with groove, melody, and rhythmic structure, participants engaged in exercises that stimulated timbral improvisation centered around thematic exploration. For example, four participants were invited to use their bodies to depict the sound of rain, accompanying a song lyric written by another participant. Through clapping, humming, whistling, and using the surfaces of percussion instruments and furniture, they created the atmosphere of a rainy evening (PN5; TV5: s3; PN6; TV6: s3; TV10: s3). Timbral improvisation also happened using drawings and collages that were created by participants and functioned as graphic scores for music-making (PR9; TV10: s2, 4; RD10; PR11; RD11). Following the creation of a drawing or collage—reflecting current emotions, self-portraits, family members, personal interests, idols, or hobbies—the group shortly discussed the drawing-as-graphic-score, identified some elements in the drawing (colors or not, forms, feelings) and engaged in timbral improvisation through bodily expressions (e.g., whistling, clapping, body percussion, voice effects) as well as unconventional use of traditional instruments, such as stroking the surface of a djembe, to create atmospheres that aligned with the drawings.

Similarly, the soundwalk—as described earlier in the result section—was also a way to shift from considering participants as performers hindered by limited instrumental skills, to participants as composers who selected meaningful sounds from their surroundings (PN8; TV8; RD8).

A fourth category of strategies aimed at mitigating language barriers. One day, a participant wrote an original lyric in Dutch about her daily activities while living in the reception center and dreams for the future. Challenges occurred when trying to find a way to put this song lyric to music. She chose not to perform in her mother tongue—concerned that the use of projected translations would divert the audience’s attention from her live performance. Instead, she preferred to write and perform in Dutch, as she wanted to learn a new language and belong to the new community. Simultaneously, she struggled with long phrases, complex pronunciations, and unfamiliar vocabulary in Dutch. Language was a barrier to convey a message and, therefore, it was important to find a way to be on the stage, feeling powerful and confident (TV5: s3; RD5). In the following rehearsal, the RF invited her to choreograph a sequence of movements inspired by her written lyrics, allowing her to communicate the essence of her message through embodied expression rather than spoken or sung language (PN6). After discussing which movements should be included, based on her song lyric, she spontaneously created a movement sequence representing her daily life in an asylum reception center. She acted as a body conductor for the RF who interpreted these movements to create accompanying music on the keyboard. When she wasn’t satisfied with the improvised music, she spoke up and explained which music would match a particular movement (TV6: s4). This excerpt from the RF’s reflexive diary demonstrates how she experienced this movement-based exercise:

It was striking how she moved effortlessly, shifting between the serenity of ritual washing and praying, to the burden of house chores, enduring the overwhelming noise t in the center, to playful moments in Dutch class. After the performance, she told me it was the first time she had ever done something like this. The weary face when she entered gave way to pure joy. Yet beneath that joy, something deeper stirred. She began to speak, haltingly at first, then with growing urgency, about the weight of the past years. She laid her heart bare before me. I had not seen it coming. This evoked in me a strong sense of responsibility as I had opened a space without being fully equipped to navigate its therapeutic implications (RD6).

Although the embodied exercise offered her a visible sense of relief, the activity also appeared to inadvertently resurface past trauma. While she expressed enjoyment and engaged deeply, the aftermath revealed emotional layers that extended beyond the scope of the session. This moment underscored the tension between roles —between music facilitation and psychological care— and left the RF feeling caught in a liminal position, unsure of the boundaries of the music practice and the ethical responsibilities it entails (RD7).

In another activity, choreographing a given lyric also appeared useful, when two participants could not sing a lyric and they were not satisfied with just speaking it (TV4: s6; TV4; TV13: s5; TV15). Incorporating movements allowed participants to experience autonomy and enjoyment by finding movements that complemented the text, thereby enabling them to express its message through means other than singing (TV13: s5; RD13). Moreover, participants felt more relaxed on stage when they could add movement to the lyrics, as they had less fear to forget the Dutch lyrics (RD15).

Finally, certain previously mentioned strategies served as a fifth category to address procedure-based challenges associated with songwriting about the difficulties of life in asylum reception centers. By employing alternative modes of expression—such as movement, soundwalks, or timbral improvisation—participants were able to communicate their messages without relying on text. This approach provided possibly a layer of protection, as it avoided the potential risks associated with overt criticism expressed through words, thereby safeguarding participants from possible repercussions (RD6).

## 5 Discussion

This study revealed place-, person- and procedure-based challenges within the songwriting workshops of *The Scratch Band*. Place-based challenges related to the multifunctional nature of the music room, with its constant flow of people, noise, and interruptions, which impeded the establishment of a safe space for creation of song lyrics. Person-based challenges related to language barriers and limited musical skills of participants, as they struggled to generate ideas for lyrics, incorporate musical elements, craft melodies from words, and ultimately perform their creations, whether through spoken or sung voice. Procedure-based challenges arose due to participants’ involvement in asylum reception procedures. The creation process was hindered by fears of repercussions for their asylum applications when translating personal experiences into lyrics, as well as by the abrupt departure of participants following the outcomes of their asylum applications.

To deal with these challenges, the RF implemented embodied strategies that combined bodily experiences in and through music (Nijs and Nicolaou, 2021; Nicolaou et al., 2023). In this qualitative study, performing bodies were conceptualized as corporeal, discursive, and agential, thereby acknowledging the body as a material presence and a site of meaning-making shaped by and shaping its surroundings. From a phenomenological perspective, the body is a body-subject or the primary site through which the world is perceived and rendered meaningful and the precondition for thought and perception (Merleau-Ponty, 1962). However, the lived realities of participants within the asylum center—made audible and visible through “moving lyrics” about daily life in the center, “Vlamingstraat 55,” and the soundwalk—reveal that the body is not merely a neutral condition for experience. Rather, following a post-structuralist framework, it emerges as “deeply contingent upon its environment and the context of the body” (Perry and Medina, 2011, p. 63). In this light, participants’ performances in the music project reflect how embodiment is modulated by the daily governance of the asylum regime, resonating with Foucault’s (1977) analysis of biopolitics and disciplinary power. His work shows how bodies are not merely biologically given, but discursively produced through regimes of knowledge, language, and normativity—rendered intelligible through institutional practices that limit movement, agency, and expression.

Yet, bodies in this project were not only shaped by their environment—they also actively shaped that environment through expressive and sonic interventions (Vallée, 2020). The soundwalk sought to bring awareness to the ambient noise of the reception center, while the moving lyrics performance offered a glimpse into the daily life of a female applicant for international protection. Her narrative—interweaving routines such as cleaning, attending language class, enduring the absence of privacy, and dreaming amid bureaucratic limbo—underscores the entanglement of the body with gender, space, and temporality. This aligns with feminist and queer theories of embodiment, which highlight how bodies are not only socially inscribed but also agential—capable of resistance, affective expression, and transformation (Butler, 1993; Grosz, 1994). This perspective reframes the body as a productive surface—a threshold rather than a container. The performer’s body is not just executing choreography or reciting lines, but converting inner memory, emotions and aspirations into outward expression. As Vallée (2020) describes, “embodiment is a mediational point of transduction between the inner resonances of the body and those resonances’ extensions into space and time that make up the singularity of place” (p. 179).

The embodied strategies in this study are not new, as similar strategies were used in applied music studies with children in general education who do not have prior music training (Hickey, 2009; Higgins and Mantie, 2013; Lampasiak and Welte, 2024; Monk, 2013), in embodied performative language pedagogy (Yeates, 2024), in sound art (Western, 2020; Keylin, 2023; Paquette and McCartney, 2012), in improvisational music therapy (Wigram et al., 2002; Bruscia, 1987; Zećo et al., 2023; Bergstrøm-Nielsen, 1993) and in aleatory music and its tradition of graphic notation, which emerged in the mid-20th century as composers sought alternatives to conventional notation systems (Bröndum, 2018). Also, in participatory music studies targeting different populations, music facilitators used comparable approaches (Petchauer et al., 2023; Dowlen et al., 2024).

The embodied turn in this study also aligns with a movement-based approach to collaborative music-making in fostering resilience, as articulated by Nijs and Nicolaou (2021). The embodied strategies in this study parallel the core principles of the authors’ theoretical and pedagogical framework, i.e., connection, exploration and experimentation, blending, cooperation, and sharing. The implemented embodied strategies align with Nijs and Nicolaou’s (2021) *connect* principle as they support engagement with (1) participants’ social and cultural context through a contemporary lens and (2) participants’ individual creative aspirations, needs, and interests, thereby fostering a collective creative space in which the boundaries between participants and music facilitators become more fluid. These embodied strategies align with the *experimentation principle* and the *blending principle* (Nijs and Nicolaou, 2021) as such activities allow letting go of rigid music structures that require certain musical skills (playing an instrument, improvise, write lyrics, and put them to music), and invite participants to freely express their ideas through moving, creating sounds, or drawing. These embodied strategies led to moments of collaborative creation and improvisation, which are examples of the *cooperation principle* and the *sharing principle* (Nijs and Nicolaou, 2021). Nevertheless, it is important to note that collaboration and sharing was not always evident within this study, due to interpersonal barriers. This aligns with previous research on gender norms in participatory music projects within asylum reception centers (Lenette et al., 2018) and with the critical observations of Beckles Willson (2021), which highlighted that forming a community through active music-making was not always the intention of participants.

What is new in this study, however, is that embodied strategies in this study were used to address specific challenges that occurred when organizing songwriting workshops in an asylum reception center.

The soundwalk functioned as a strategy to initiate dialog around place-based challenges. In accordance with Hildegard Westerkamp’s delineation of sound walking’s core functions—*orientation, dialog, and composition* (McCartney, 2000, p. 4)—this practice enabled a critical and embodied engagement with the auditory textures of place. Through processes of attentive listening and spatial movement, knowledge emerged from the ephemeral interplay of sound and activity. The resulting sound map functioned not only as an interpretive artifact but also as a communicative medium, intended to inform, sensitize, and provoke reflection on the daily realities of life within the center. Moreover, it introduced a creative modality of narrative representation (Westerkamp, 1994), reconfiguring the sonic environment into a space of meaning-making. By recomposing the sonic environment into a musical artifact, the participant emphasized not only its transience but also its potential for transformation—rendering them, in Foreman’s (2010, p. 9) words, “dwelling places; both places of exile and places to emigrate to.”

Through engagement in timbral improvisation activities, soundwalk or moving a lyric instead of speaking, participants could select the themes they wished to elaborate on or convey their messages without words, thereby countering procedure-based challenges. Not all participants considered storytelling as safe, which counters the common argument in existing literature that songwriting workshops would effortlessly “amplify unheard voices” (Birch, 2023; Gallo and Kuchenbrod, 2022; Mani, 2022), enable participants to “reflect on past experiences” (Cohen and Henley, 2018), and “challenge dominant negative narratives” (Hess, 2018, p. 7). Whether this fear is substantiated remains unknown; it appears less as a reaction to specific threats within the current context and more as a deeply internalized caution—an unwillingness among participants to take risks, perhaps rooted in past experiences were speaking out carried severe consequences, including imprisonment. Regardless of whether such fears are factually grounded, acknowledging them is essential, as the creation of a genuinely safe and trusting space is a foundational ethical obligation in this kind of participatory work.

Furthermore, detecting person-based challenges such as different (musical) interests, limited inspiration, musical skills, demonstrates that it is not always easy to tap into “innate capacity for being creative and playful in their day-to-day life” (Mani, 2022, p. i37) or foster self-expression and enjoyment (Cohen and Wilson, 2017). Not all participants necessarily possess songwriting or musical aspirations and found greater comfort in performing familiar repertoire or making visual art. This notion aligns with previous studies that provide insights into music projects with newly arrived migrants and asylum seekers and highlight the fact that well-known songs can take on and convey new meanings in these contexts, as well as well as providing a source of comfort (Nunn, 2018; Lenette, 2019; Sunderland et al., 2015). Moreover, songwriting was a less accessible activity than initially assumed, as participants had limited inspiration or musical skills. Implementing embodied strategies enabled participants to take part in unpredicted ways, thereby creating more effective learning environments.

This way of working resonates with the constraint-led approach. In this pedagogical approach, constraints are strategies designed to minimize ineffective engagement with tasks (Abrahamson and Sánchez-García, 2016). Constraints define what learners can do while also fostering openness to possibilities, enabling them to discover task-specific solutions (Hopper, 2012). The embodied strategies used in this action research align with the constraints (environmental, individual and task) defined by Newell (2003). For example, organizing a soundwalk and incorporating the noise of an asylum reception center into a music composition can function as an environmental constraint. In this case, replacing musical instruments with environmental sounds and noise involves manipulating the available materials (environmental constraints) to engage in composition. Reimagining traditional approaches to music-making and introducing new activities such as creating drawings and bringing them to life through embodied improvisation instead of writing song lyrics and composing songs, facilitating timbral explorations rather than focusing on tonal improvisation with musical instruments, and narrating their stories through movement instead of performing lyrics in an unfamiliar language, involve manipulating task constraints by modifying the goals or conditions associated with a task (Rosengren and Braswell, 2003; Hopper, 2012).

When verbal expression felt unsafe, non-verbal modes of communication were embraced to convey critical messages, thereby modifying individual constraints. The latter were also manipulated by introducing new roles: from “instrument players,” “tonal improvisers” “language learners” in cycle 1 to “composers,” “timbral improvisers” and “body conductors” in cycle 2. This shift in roles demonstrates that participants can have different roles in one project, and how —in alignment with Bronfenbrenner and Morris (2006)—these new roles create new expectations. In this way, the musical workshop, here framed an act of hospitality, could work and welcome people, irrespective of limited inspiration or skills (Higgins, 2012), and was responsive, as it not only embraced the preferences of participants, but also their needs and strengths (Kenny, 2022).

Although the above insights are important and useful, it is also important to mention the study’s limitations. A first limitation concerns the multiple roles the RF was asked to play, and particularly the risk that embodied strategies may re-open trauma in contexts lacking adequate support. In this project, responsibilities extended beyond those of a music facilitator and organizer, at times resembling the roles of a music therapist or a social worker—areas in which she was not trained. Many refugees and asylum seekers live with complex trauma, stemming from experiences of violence, persecution, and systemic instability in their countries of origin, compounded by post-migratory stressors such as social isolation, insecure legal status, and institutional alienation (Filges et al., 2024; Mulcaire et al., 2024). As trauma is deeply embodied and recovery involves the integration of mind and body (Van der Kolk, 2014), embodied art-based practices can hold therapeutical potential by engaging affective and sensory experiences. While this project was not intended as music therapy and with participants not treated as clients, it nonetheless, posed risks related to trauma exposure and emotional vulnerability. The absence of a formal “trauma-informed” framework (Harrison et al., 2019), combined with environmental limitations—such as constant noise, movement, and surveillance within the reception center—rendered some situations ethically and emotionally complex. Encounters with participants’ psychological distress underscored the challenges of maintaining professional and emotional boundaries in a setting where support infrastructures are limited.

The second limitation relates to the small scale of the study and the non-universality of embodied strategies. While this study demonstrated that embodiment musicking was beneficial for some participants, it examined only one facilitation process. As the sample scale is rather small and focuses on one specific context, generalized conclusions suggesting transferability to music projects in asylum centers broadly cannot be made. Moreover, embodiment musicking was not equally embraced by all. Some individuals chose not to engage in bodily-based exercises. Embodiment is not a universally effective or context-neutral approach; its efficacy depends on the individuals involved, the social and political environment, and the relational dynamics at play. What resonated here may not resonate elsewhere. Embodiment, therefore, should not be romanticized as a universally applicable solution; rather, it should be considered one tool among many in a facilitator’s repertoire—offered selectively, responsively, and with participant consent.

A third limitation is epistemological and methodological. As the primary narrator of this project, the RF was the one writing about the participants’ embodied experiences. Yet, as Ellingson (2017) notes, participants’ bodies cannot be understood apart from the language through which they are interpreted. Any account the RF provides is necessarily filtered through her own cultural and linguistic frameworks, and thus, her writing cannot claim to report unmediated bodily experience. Also, in terms of methodology, community involvement was not present in each iterative design phase. For the design of music interventions, the RF did not consult participants, but designed it herself. Although participants could give their opinion on the proposed intervention, they were not invited to propose interventions themselves. This may have limited the intervention’s relevance and participant’s ownership.

## 6 Conclusion

This study revealed that the complex interplay of place-, person- and procedural-based challenges significantly shaped both music creation processes and group dynamics within *The Scratch Band* project. The findings underscore that, while songwriting holds transformative potential, its implementation in the specific context of a Belgian asylum reception center demanded a high degree of critical reflexivity, adaptability from the facilitator.

This study contributes to the existing literature by examining how embodied strategies—such as a soundwalk, embodied musical brainstorms, timbral improvisation, and moving lyrics—could (partially) address the challenges faced when facilitating songwriting in an asylum reception center. These embodied strategies offered alternative entry points into songwriting, circumventing linguistic, musical, and psychological barriers. In doing so, they reimagined participants not only as applicants for international protection or musical instrument and language learners, but in different roles as composers, storytellers, and agents of sonic citizenship. This aligns with the constraint-led approach to facilitation and provides a more inclusive and responsive way of facilitating participatory music-making. The findings also surface important tensions related to trauma, power, and the ethics of facilitation, pointing to the need for continued interdisciplinary collaborations and trauma-informed research into participatory music practices in humanitarian contexts.

Future research might further investigate how the constraint-led approach can be adapted to transient environments such as asylum reception centers. This study suggests that consciously integrating the constraint-led approach in response to occurring challenges, is a valuable avenue for further exploration. Moreover, future research can explore ways to involve community dialog in each phase of the project, for instance in the co-design of music interventions, in view of increasing participants’ ownership.

In terms of implications for the field practice, this study provides music facilitators with concrete, embodied strategies that have shown value in navigating the complex realities of facilitation in an asylum reception center. These approaches may inspire music facilitators to diversify their methods when facing similar linguistic, psychological, and contextual barriers. The growing presence of participatory music practices in asylum reception centers underscores the need for equipping facilitators with the necessary tools to navigate these complex and precarious settings. As such, the integration of embodied strategies and the constraint-led approach, is suggested as valuable addition to facilitator training programs, with the aim of contributing to ongoing professional development within the field.

## Data Availability

The datasets presented in this article are not readily available because they contain information about residents of an asylum reception center. Questions regarding the datasets should be directed to the corresponding author.
